# Sox9 Expression in the Second Heart Field; A Morphological Assessment of the Importance to Cardiac Development with Emphasis on Atrioventricular Septation

**DOI:** 10.3390/jcdd9110376

**Published:** 2022-11-02

**Authors:** Raymond N. Deepe, Jenna R. Drummond, Renélyn A. Wolters, Emily A. Fitzgerald, Hannah G. Tarolli, Andrew B. Harvey, Andy Wessels

**Affiliations:** Department of Regenerative Medicine and Cell Biology, Medical University of South Carolina, Charleston, SC 29425, USA

**Keywords:** development, septation, congenital heart defect, Sox9, second heart field

## Abstract

Failure to form the septal structures that separate the left and right cardiac chambers results in defects that allow shunting of blood from one side of the heart to the other, leading to the mixing of oxygenated and de-oxygenated blood. The atrioventricular (AV) mesenchymal complex, consisting of the AV cushions, the Dorsal Mesenchymal Protrusion (DMP), and the mesenchymal cap, plays a crucial role in AV septation. Cells found in these structures derive from different cell lineages. In this study we have investigated the role of the transcription factor Sox9 in the Second Heart Field (SHF) with the emphasis on the formation of the atrioventricular septal complex. Using a mouse model in which Sox9 is conditionally deleted from the SHF we demonstrate that in this model virtually all mouse embryos develop septal abnormalities, including complete atrioventricular septal defects (cAVSDs) and isolated ventricular septal defects. Our morphological analyses indicate that perturbation of the development of the mesenchymal cap appears to play a crucial role in the pathogenesis of the atrial septal defects observed in the AVSDs and suggests that this component of the AV mesenchymal complex might play a more important role in AV septation than previously appreciated.

## 1. Introduction

The Second Heart Field (SHF) is a population of cardiac progenitor cells that after the initial formation of the First Heart Field (FHF)-derived primary heart tube contributes to the developing heart. At the arterial pole, the SHF contributes to the outflow tract (OFT), the right ventricle (RV), and the ventricular septum (VS) while at the venous pole, the SHF contributes to a subset of cells in the atrial myocardium and the dorsal mesenchymal protrusion (DMP) and its derivatives [1]. The importance of the SHF for normal heart development has been demonstrated in a variety of studies in which perturbation of gene expression in the SHF resulted in heart defects [2,3,4]. 

Atrioventricular Septal Defects (AVSDs) are serious heart malformations found in 5% of all patients with congenital heart disease (CHD) [5,6]. They are particularly prevalent in genetic disorders such as Down syndrome (20-25%) and CHARGE syndrome (13%) [7,8,9]. The two major forms of AVSDs are the complete AVSDs (cAVSD) and the incomplete/partial AVSDs (pAVSD). A cAVSD is characterized by the presence of a primary atrial septal defect (pASD), a ventricular septal defect (VSD), and a common AV valve (cAVV). In contrast, a pAVSD typically has an pASD in combination with a cAVV in the absence of a VSD [5,10]. In this paper, we will refer to this latter form of pAVSD as an pAVSD/pASD. A less common form of partial AVSD is the one where shunting only takes place at the ventricular level through a ventricular septal defect (VSD) [5,11,12]. In this paper, we will refer to this form as pAVSD/VSD.

Atrioventricular septation is critically dependent on the proper formation of the AV mesenchymal complex. This complex initially consists of four separate entities; the two endocardial-derived major AV cushions, the mesenchymal cap on the developing primary atrial septum, and the Dorsal Mesenchymal Protrusion (DMP). These components eventually fuse together to separate the two atrial chambers and contribute to the formation of the valvuloseptal complex [3,10,13,14,15]. 

Historically, the structural defects observed in AVSDs were believed to result from abnormal development of the AV cushions. These malformations were therefore commonly referred to as “endocardial cushion defects” [16,17,18,19,20]. In recent years, the insight into the pathogenesis of AVSDs has significantly changed. Studies from a number of different groups have implicated abnormal development of the SHF-derived DMP in the pathogenesis of pASDs, i.e., the most common single septal defect in virtually all forms of AVSDs [2,3,4,10,13,14,21,22,23]. Understanding of the molecular mechanisms that govern DMP development has steadily developed over the last 10 years. Genes that have been identified as playing a critical role in DMP development include Alk3/BmpR1a [3], Smoothened (Smo) [2,4,23], Wnt2 [24], Wnt11 [25], Shox2 [26], and Tbx5 [27].

Sox9 (SRY-type box 9) is a transcription factor that has an important function in the development of the AV cushions and valve formation [28,29,30,31]. Based on earlier observations [2], and after revisiting the published literature, we developed the hypothesis that Sox9 could play a role in SHF-dependent development of the AV valvuloseptal complex. In this study, we describe the spatiotemporal expression of Sox9 as it relates to the contribution of the SHF to the AV mesenchymal complex. Our data suggest that SHF-specific deletion of Sox9 does not significantly affect the development of the AV cushions, nor that of the DMP, but that it instead leads to a significant perturbation in the development of the mesenchymal cap. Half of the embryonic offspring of the mouse model in which Sox9 is deleted from the SHF develop cAVSDs. Interestingly, an isolated VSD was observed in the rest of the embryonic offspring. Analysis of specimens in which Sox9 is deleted from the SHF indicates that these VSDs are associated with a developmental perturbation of the development of the proximal part of the embryonic outlet septum. Combined, our results show that the expression of Sox9 in the SHF is critically important for proper cardiac septation and that Sox9 might play a more extensive role in AV valvuloseptal morphogenesis than previously recognized.

## 2. Materials and Methods

### 2.1. Mice

The Mef2c-AHF-cre mouse model (in the text referred to as SHF^cre^ ) was kindly provided by Dr. Brian Black [32]. The Tie2-cre (Tie2^cre^) mouse was initially provided by Dr. Yanagisawa [33](available from the Jackson laboratory under B6.Cg-Tg(Tek-cre)1Ywa/J), and the Nfatc1-cre (Nfatc1^cre^) by Dr. Bin Zhou [34]. The STOCK Smo^tm2Amc/J^ (floxed Smo) mouse, the B6.129S7-*Sox9^tm2Crm^*/J (floxed Sox9) mouse, and the B6.129(Cg)-Gt(ROSA)26Sor^tm4(ACTB-tdTomato,dTomatLuo/J^ (R26^mG^ reporter expressing GFP ) mouse were all obtained from the Jackson Laboratory. Generation and use of all mouse models has been described previously [32,33,34]. To trace the fate of cells derived from the SHF, the Mef2c-AHF-cre mouse was used in combination with the ROSA26^mT/mG^ reporter mouse. To trace the fate of endocardially derived cells the Tie2^cre^ and Nfatc1^cre^ mice, respectively, were used in combination with the R26^mT/mG^ mouse essentially as previously described [3]. The SHF^cre^;Smo^fl/fl^ conditional knockout mice used in this study were generated in the context of an earlier study [2]. For the generation of SHF^cre^;Sox9^fl/fl^ conditional knockout, male SHF^cre^;Sox9^fl/+^ mice were mated with female Sox9^fl/fl^ or Sox9^fl/+^ mice. In many (but not all) cases we also included the R26^mG^ allele in the crosses. This created an offspring where a subset of specimens would express SHFcre-driven GFP expression in SHF-derived cells. To simplify the description of the offspring in the respective mating protocols, we include “(R26^mG^)” in the group name, indicating that not all specimens were carrying the R26^mG^ allele. These mating regimens produced SHF-specific homozygous and heterozygous Sox9 knockout mice and control littermates at stages ranging from ED9-P0. All experiments using animals were approved by the MUSC Institutional Animal Care and Use Committee (IACUC) under protocol ID number IACUC-2020-01140 and complied with all federal and institutional guidelines. 

### 2.2. Histology

Following the sacrifice of time-pregnant dams, embryos were isolated in phosphate-buffered saline (PBS) and inspected using a dissecting microscope to establish developmental stage. Embryos were then fixed overnight in freshly dissolved paraformaldehyde (4% *w*/*v* in PBS), processed through a graded series of ethanol, cleared in toluene, embedded in Paraplast (Leica, catalogue#: 39602004), serially sectioned (5 μm), mounted on Unifrost Plus microscope slides (Azer Scientific catalogue#: EMS200W+) and stored at room temperature. Hematoxylin/eosin staining was performed as previously described [1,15,35].

### 2.3. Immunofluorescent Antigen Detection

Immunofluorescence was performed as previously described [2,3,36] with antibodies recognizing the following antigens: Myosin Heavy Chain (DSHB; MF20), Sox9 (Novus; NBP1-85551), EGFP (Aves Lab; GFP-1020), and Islet 1 (R&D; AF1837). Secondary antibodies (Jackson Immunoresearch) included anti-chicken FITC (703-095-155), anti-mouse TRITC (715-025-150), anti-goat TRITC (705-025-147), and anti-rabbit Cy5 (711-605-152). Nuclei were visualized using DAPI (Invitrogen; Slowfade Gold Antifade Reagent with DAPI; catalogue#: S36938) and fluorescence visualized using a Zeiss AxioImager II microscope.

### 2.4. AMIRA 3D Analysis

5 µm serial sections from SHF^Cre^;Sox9^fl/fl^;R26^mG^ and SHF^Cre^;Sox9^fl/+^;R26^mG^ specimens were immunofluorescently stained and imaged on a Zeiss AxioImager II microscope. From these images, reconstructions and volume statistics of these sections were generated using Amira 3D software. 

### 2.5. Cell Profiler Analysis

5 µm serial sections from SHF^Cre^;Sox9^fl/fl^;R26^mG^ and SHF^Cre^;Sox9^fl/+^;R26^mG^ specimens were immunofluorescently stained and imaged on a Zeiss AxioImager II microscope. Images were then edited for areas of interest and cell counts were then generated from these regions using Cell Profiler 3.1.8 software to identify and count DAPI stained nuclei.

### 2.6. Statistical Analysis

Quantitative data were statistically analyzed using unpaired *t*-test. *p*-values of less than 0.05 were considered not significant.

## 3. Results

### 3.1. Sox9 Expression in the Mesenchymal Tissues Contributing to the Atrioventricular Valvuloseptal Complex

Sox9 is involved in several aspects of cardiac development. It is particularly well-studied in the context of formation of the AV cushions and AV valve development [28,29,30,31]. Previously, it has been reported that deleting Sox9 from the endocardial cell lineage leads to hypoplasia of the AV cushions and malformations at the AV junction. In addition to figures that demonstrate cushion/valve abnormalities, some figures also show that the experimental mice develop septal defects that resemble the abnormalities reported in mouse models in which genes important for the proper development of the SHF are deleted [2,3,4,10,24]. This prompted us to investigate the role of Sox9 in the SHF and to determine whether it is involved in the development of septal structures at the cardiac venous pole. 

To obtain a comprehensive insight into the pattern of Sox9 expression within the SHF and in the respective components of the AV mesenchymal complex, we immunofluorescently stained serial sections of embryonic offspring of wildtype mice and embryos generated by crossing the SHF-specific Mef2c-AHF-cre mouse [33] (referred to as SHF^cre^ in this publication) with the B6.129(Cg)-Gt(ROSA)26Sor^tm4(ACTB-tdTomato,dTomatLuo/J^ reporter mouse (R26^mG^ hereafter). In SHF^cre^;R26^mG^ embryos, the SHF is characterized by the expression of GFP [2,3]. The primary focus of this study was to determine the role of Sox9 in the SHF in relation to the development of the AV valvuloseptal complex. We, therefore, focused primarily on the relationship between the cells found in the posterior SHF (pSHF) and the expression of Sox9 in and around this cell population. 

#### 3.1.1. Embryonic Day (ED) 9.5

**Figure 1:**
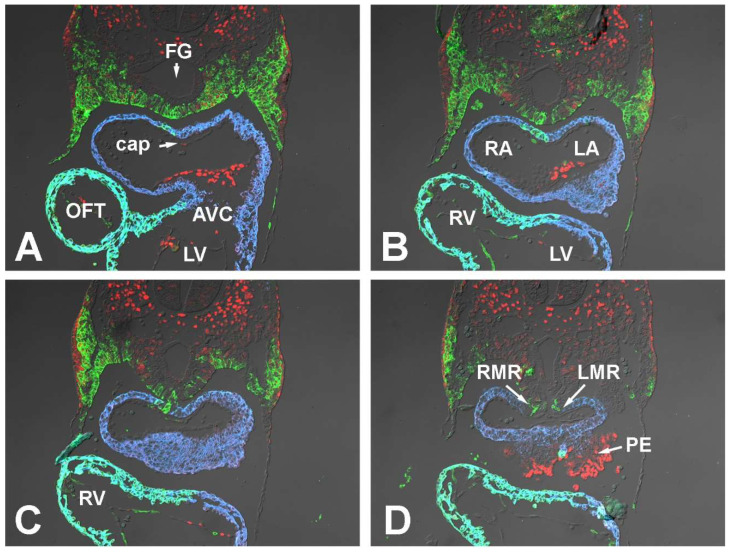
Sox9 expression at embryonic day 9.5. Panels (**A**–**D**) show serial sections of transversely sectioned SHFcre;R26mG embryos at ED9.5 immunofluorescently stained for the presence of Sox9 (red), Myosin Heavy Chain (blue), and GFP (green). The GFP staining delineates the SHF and SHF-derived cells. AVC = atrioventricular cushion, cap = mesenchymal cap, FG = foregut, PE = proepicardium, OFT = outflow tract, LA = left atrium, LMR = left mesocardial reflection, LV = left ventricle, RA = right atrium, RMR = right mesocardial reflection, RV = right ventricle.

**Figure 2:**
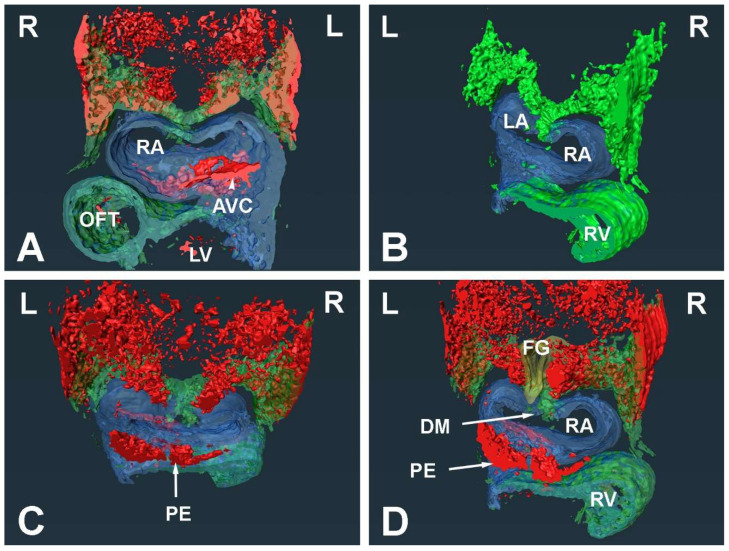
Three-dimensional reconstruction of the spatial distribution of Sox9 in the posterior SHF. Serial sections of an ED9.5 SHFcre;R26mG heart were immunofluorescently stained for Sox9 (red), Myosin Heavy Chain (blue), and GFP (green). In (**A**–**D**) the respective staining patters were 3-dimensionally reconstructed using AMIRA-3D software. AVC = atrioventricular cushion, DM = dorsal mesocardium, FG = foregut, PE = proepicardium, OFT = outflow tract, LA = left atrium, LV = left ventricle, RA = right atrium, RV = right ventricle.

To map the spatial distribution of pSHF cells in ED9.5 SHF^cre^;R26^mG^ embryos, we fluorescently stained transversely sectioned specimens for the presence of GFP. This showed that in the embryonic midline at the level of the developing atria pSHF cells are mainly concentrated in the area between the foregut and the heart. From here, the pSHF domain fans out laterally toward the body walls (Figure 1A,B and Figure 2). At the caudal-most end of the heart, i.e., the sinoatrial junction (Figure 1C,D and Figure 2), very few GFP positive cells are seen. The 3D reconstructions generated based on these stainings (Figure 2) clearly visualize how the GFP-expressing domain of pSHF cells tapers off toward the venous pole, where only a small number of SHF cells are found in the mesocardial reflections of the dorsal mesocardium (Figure 2D). 

Within the pSHF, Sox9 is expressed most prominently in the cell population that is located between the foregut and the body walls (Figure 2). The cells at the dorsoventral midline, including the SHF cells representing the precursor population of the DMP, express relatively little Sox9 at this stage (Figure 1A–D and Figure 2A,C,D). 

At ED9.5, the individual components of the AV mesenchymal complex are either in the early phases of their development (e.g., the AV cushions and mesenchymal cap) or have yet to form (e.g., the DMP). In the developing AV cushions, Sox9 expression is seen in the endocardial lining and in the endocardial-derived cells (ENDCs) that have started to invade and populate the cushions (Figure 1A,B). Although there is no sign yet of a developing primary atrial septum, the local accumulation of extracellular matrix (ECM) in the roof of the common atrium demarcates the spot from where the primary atrial septum (pAS) will emerge in subsequent stages (Figure 1A). This ECM-filled space will eventually become the mesenchymal cap on the leading edge of the pAS. A few Sox9 positive cells can be seen in the endocardial lining of the developing cap, but the majority of the endocardial cells do not express Sox9 (Figure 1A). Given our long-standing interest in the role of the SHF-derived DMP, we paid extra attention to the expression of Sox9 in the pSHF cells in and around the dorsal mesocardium as that is the location from where the proliferating population of pSHF cells will expand into the common atrium and give rise to the DMP. Our study showed that there is virtually no Sox9 expression in the pSHF cells in the vicinity of the dorsal mesocardium (Figure 1C,D and Figure 2A,C,D). 

#### 3.1.2. Embryonic Day 10.5

**Figure 3:**
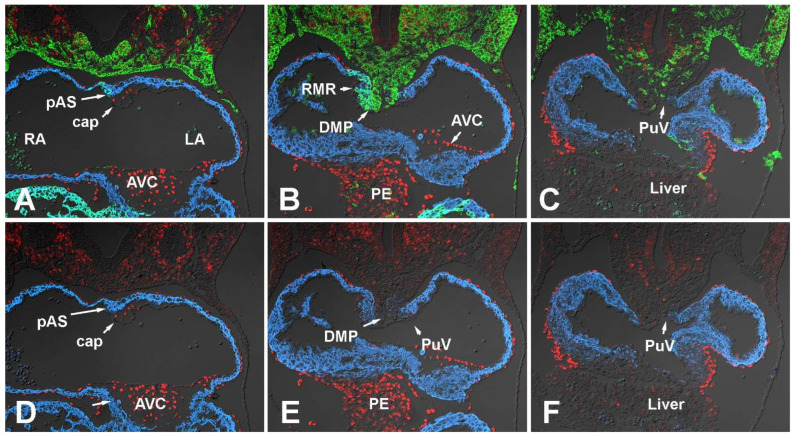
Sox9 expression at embryonic day 10.5. Panels (**A**–**C**) show serial sections of embryonic SHFcre;R26mG hearts at ED10.5 immunofluorescently stained for the presence of Sox9 (red), Myosin Heavy Chain (blue), and GFP (green). The GFP staining delineates the SHF and SHF-derived cells. In (**D**–**F**), only the Myosin Heavy Chain (blue) and Sox9 (red) are shown to more clearly show the Sox9 distribution. AVC = atrioventricular cushion, cap = mesenchymal cap, DMP = dorsal mesenchymal protrusion, PE = proepicardium, LA = left atrium, LV = left ventricle, pAS = primary atrial septum. PuV = pulmonary vein, RA = right atrium, RMR = right mesocardial reflection, RV = right ventricle.

When compared to ED9.5, the general distribution pattern of SHF-derived cells and the expression of Sox9 at the venous pole of the heart has not significantly changed (Figure 3A–C). Sox9 expression is still found in the lateral most aspects of the GFP-positive pSHF. Still very little Sox9 expression is observed in the pSHF cells located in the pSHF tissues associated with the dorsal mesocardium (Figure 3B,C,E,F). 

Within the respective components of the AV mesenchymal complex, the expression of Sox9 resembles that of ED9.5. Sox9 is expressed in a small number of endocardial cells lining the AV cushions as well as in the endocardial-derived cells that have populated these cushions (Figure 3A,D). A small myocardial ridge in the roof of the common atrium represents the emerging pAS (Figure 3A,D) and the small cushion-like structure developing in association with the pAS is the developing mesenchymal cap. This cap will eventually run along the leading edge of the pAS in later stages of development. A few cells in the endocardial lining of the cap express Sox9 (Figure 3A,D). In addition, all mesenchymal cells located within the cap express Sox9 as well. Earlier cell fate studies with endocardial-specific Tie2^cre^ mouse have demonstrated that the mesenchyme of the cap largely derives from endMT of the endocardial lining of the cap [14,15,37]. It is important to point out that if an SHF-derived endocardial cell would undergo endMT, this mesenchymal cell would be GFP-labeled in a Tie2^cre^;R26^mG^ mouse and would, solely based on this lineage trace approach, be indistinguishable from endocardially derived mesenchymal cells that are not derived from the SHF. 

The DMP develops between ED9.5 and ED10.5 when a subpopulation of pSHF cells expands into the common atrium using the dorsal mesocardium as a portal of entry [3,10,13,14,15]. As shown in Figure 3B,E, this process also involves the inward folding of the right mesocardial reflection (RMR) into the future right atrium which is by and large a myocardial structure consisting of SHF-derived cells. The splanchnic mesoderm/pSHF that is located between the foregut and the dorsal mesocardium as well as the pSHF cells that form the DMP express very little Sox9 at this stage (Figure 3B,C,E,F).

Even though the proepicardium (PE) is not part of the AV mesenchymal complex, it needs to be noted that Sox9 is expressed in the PE and in the epicardially derived cells (EPDCs) that derive from the PE and start to migrate over the myocardial surface around this time [38]. At the AV junction, EPDCs will eventually contribute to a subset of leaflets of the AV valves [39].

#### 3.1.3. Embryonic Day 11.5

**Figure 4:**
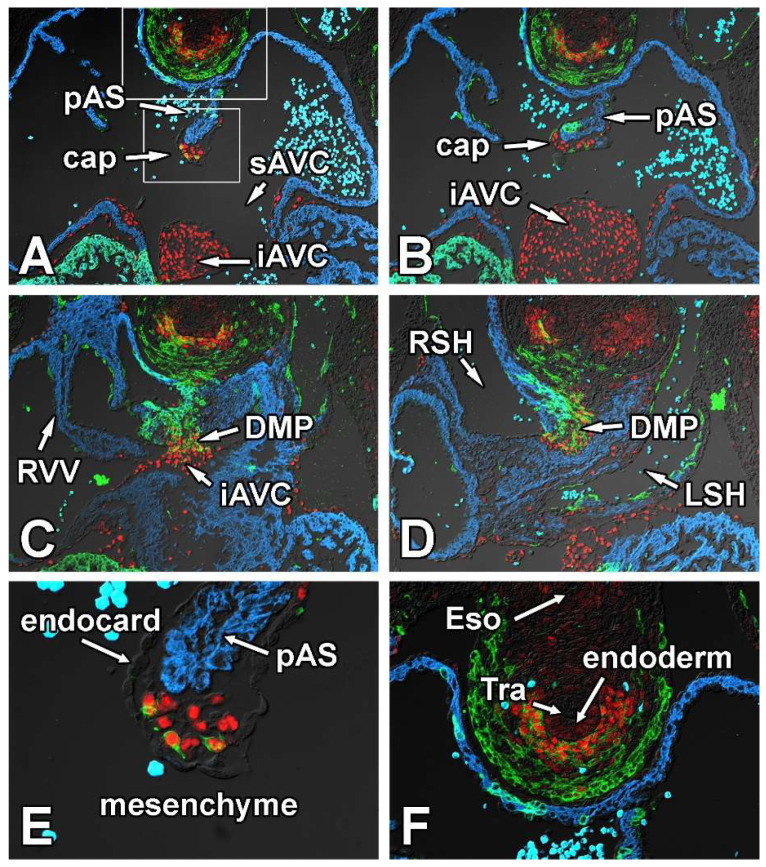
Sox9 expression at embryonic day 11.5. Panels (**A**–**F**) show the expression of Sox9 (red), Myosin Heavy Chain (blue), and GFP (green) in SHFcre;R26mG embryos at ED11.5. The GFP staining delineates the SHF and SHF-derived cells. Panels (**E**,**F**) are enlargements of the boxed areas in Panel (**A**). Note the GFP expressing mesenchymal cells within the cap (**A**,**E**). AVC = atrioventricular cushion, cap = mesenchymal cap, DMP = dorsal mesenchymal protrusion, endocard = endocardium, eso = esophagus, iAVC = inferior AV cushion, LA = left atrium, LSH = left sinushorn, pAS = primary atrial septum, RA = right atrium, RSH = right sinushorn, RVV = right venous valve, sAVC = superior AV cushion, Tra = trachea.

**Figure 5:**
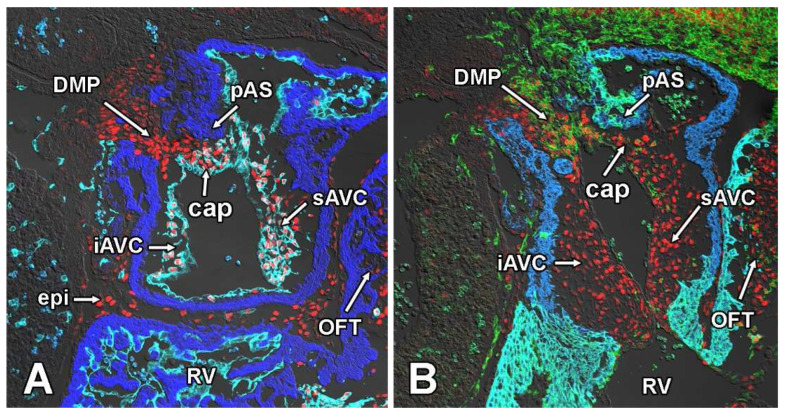
Sox9 expression in the mesenchymal cap and DMP at embryonic day 11.5. Panels A and B show sagittal sections of Nfactc1cre;R26mG (**A**) and SHFcre;R26mG (**B**) embryonic hearts at ED11.5. The section in panel (**A**) was stained for GFP (light green) to delineate endocardial and endocardiallyderived cells, Myosin Heavy Chain (dark blue), and Sox9 (red). The section in panel (**B**) was stained for GFP (green) to delineate SHF and SHF-derived cells, Myosin Heavy Chain (blue), and Sox9 (red). Both sections show how the respective components of the AV mesenchymal complex (cap, AV cushions, and DMP) are contiguous and how the Sox9 expressing mesenchyme of AV cushions and cap is endocardially derived (**A**). The SHF-derived mesenchyme of the DMP expresses Sox9 at this stage (**B**) cap = mesenchymal cap, DMP = dorsal mesenchymal protrusion, epi = epicardium, iAVC = inferior AV cushion, sAVC = superior AV cushion, OFT = outflow tract, pAS = primary atrial septum, RV = right ventricle.

The analysis of embryos at ED11.5 provides a good insight both into the spatial relationship of the respective tissues that contribute to the AV mesenchymal complex and gives valuable information about the spatial expression pattern of Sox9 in the cardiac and extra-cardiac tissues associated with the developing venous pole of the heart. For this part of the study, we serially sectioned SHF^cre^;R26^mG^ embryos in both the transverse (Figure 4) and sagittal (Figure 5) planes and stained for the presence of GFP, Sox9, and Myosin Heavy Chain. 

The expression of Sox9 in the pSHF located between the trachea (a derivative of embryonic foregut) and the heart is strongest in cells located immediately ventral to the trachea (Figure 4A–C,F). Most, if not all, endocardial-derived mesenchymal cells in the AV cushions and the mesenchymal cap express Sox9 (Figure 4A,B and Figure 5A,B). As a result of the complex remodeling that is taking place at the caudal-most part of the dividing atria, it is difficult to identify in transverse sections the DMP as a stand-alone structure as it has fused with the cap and the inferior AV cushion (Figure 4C,D). However, in specimens that are sectioned in a sagittal plane the DMP is still easily identified (Figure 5A,B). Whereas the pSHF-derived mesenchymal cells in the DMP expressed little Sox9 up to ED10.5 (see above), the level of expression at ED11.5 is relatively high and, based on intensity of fluorescent antibody labeling, comparable with that observed in the ENDCs of the AV cushions and the cap (Figure 4C,D and Figure 5A,B). In transverse sections, occasionally one or more GFP positive and Sox9-expressing cells can be found in the cap (Figure 4A,E). While it is hypothetically possible that these are SHF-derived cells that have migrated from the DMP into the cap, the fact that we have not observed any GFP-negative mesenchymal cells in the cap when lineage tracing was conducted with the Tie2^cre^;R26^mG^ mouse, makes it highly unlikely that that is the case.

### 3.2. Conditional Deletion of Smoothened from the SHF Results in Downregulation of Sox9 in the pSHF

As previously demonstrated, the Hedgehog (Hh) and Wnt/ß-catenin signaling pathways are critically important in the regulation of the development of the SHF and in AV septation [2,24,25]. Both pathways are also involved in regulating Sox9 expression [40,41,42,43,44,45]. In earlier studies, we reported that SHF-specific deletion of Smoothened (Smo), a transmembrane protein and signaling mediator in the Hh pathway, led to the reduction of proliferation of cells in the pSHF and the inhibition of DMP development. This ultimately resulted in AVSDs in the SHF^cre^;Smo^fl/fl^ offspring [2]. In addition, we observed that the SHF-specific Smo deletion led to reduced Wnt/β-catenin signaling in the pSHF. Since Sox9 can be regulated by both the Hh and Wnt/β-catenin pathways, we sought to determine whether, and if so how, Sox9 expression was affected in the SHF^cre^;Smo^fl/fl^ mouse. 

**Figure 6:**
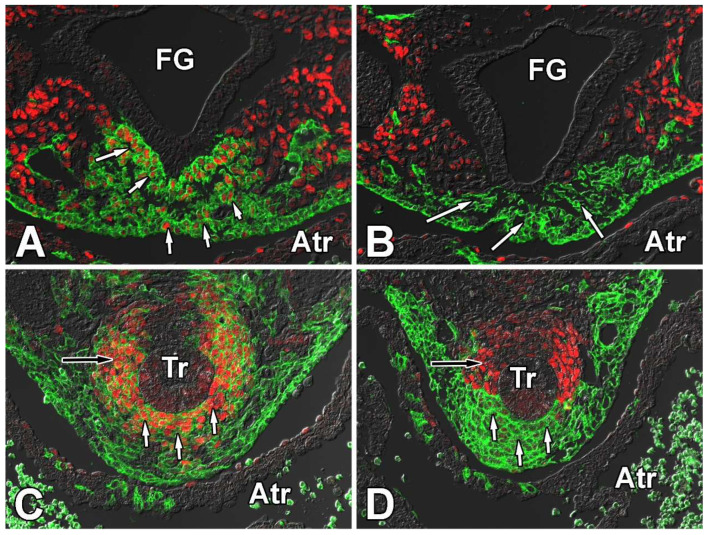
Deletion of Smoothened from the SHF results in downregulation of Sox9. Panels (**A**,**B**) show the expression of Sox9 (red) in relation to the SHF (green) at ED10.5 ventral to the developing foregut, while (**C**,**D**) show the situation in the same area at ED11.5. The sections in (**A**,**C**) are from SHFcre;R26mG control specimens showing the expression of Sox9 in a subset of the GFP-labeled SHF cells, whereas (**B**,**D**) are sections from SHFcre;Smofl/fl;R26mG embryos demonstrating the fact that deletion of Smo from the SHF has resulted in loss of Sox9 from these cells as well. Atr = atrium, FG = foregut, Tr = trachea.

In ED10.5 and ED11.5 SHF^cre^;Smo^fl/fl^;R26^mG^ specimens, a pronounced reduction was seen in the level of Sox9 expression in the pSHF when compared to SHF^cre^;Smo^fl/+^;R26^mG^ controls (Figure 6A–D), specifically in cells located ventral to the foregut endoderm. As the ventral foregut endoderm secretes Sonic Hedgehog (Shh) [46] and Sox9 expression can be regulated by Shh [37], this suggests that, within the pSHF, Sox9 is a downstream target of the Hh signaling pathway.

### 3.3. Creating a SHF-Specific Sox9 Knockout Model to Study Sox9 Dependent Development of the AV Valvuloseptal Complex

The widespread expression of Sox9 in the tissues that contribute to the AV mesenchymal complex in combination with the reduction of Sox9 in the SHF in the SHF^cre^;Smo^fl/fl^ mouse at stages critical to the development of the formation of the AV mesenchymal complex, prompted us to further investigate the importance of Sox9 expression in the SHF. To this end, we developed a SHF-specific Sox9 knock-out model by crossing the Mef2c-AHF-cre (SHF^cre^) mouse with mice carrying floxed Sox9 alleles. In many, but not all, cases, we included in the crosses also the Rosa26^mT/mG^ (R26^mG^) allele, thus creating an offspring where a subset of specimens would carry the R26^mG^ reporter allele. To simplify the description of the offspring in the respective mating protocols, we include in this paper a [R26^mG^] symbol in the group name to indicate that specimens from these mating protocols may or may not carry the R26^mG^ allele. Two different mating protocols were used to generate mice with a significant number of complete SHF-specific Sox9 knockouts. Using this strategy, we collected for this study 250 embryos from SHF^cre^;Sox9^fl/+^xSox9^fl/fl^;[R26^mG^] crosses and 45 embryos from SHF^cre^;Sox9^fl/+^xSox9^fl/+^;[R26^mG^] crosses between ED9 and ED18.5. From these approaches, we collected in total 63 SHF^cre^;Sox9^fl/fl^;[R26^mG^] specimens (i.e., SHF-specific Sox9 knockouts), the remaining specimens did either not express the cre construct and/or carried only one Sox9 floxed allele. No viable postnatal specimens were recovered. All SHF^cre^;Sox9^fl/fl^;[R26^mG^] were either stillborn or died shortly after birth. Assessment of the genotypes of the embryonic offspring showed expected Mendelian ratios during the embryonic stages and up to birth, indicating that deleting Sox9 from the SHF did not lead to prenatal lethality. The cause of the peri/postnatal death has not yet been determined.

### 3.4. Second Heart Field-Specific Deletion of Sox9 Results in AVSDs and Isolated VSDs

**Figure 7:**
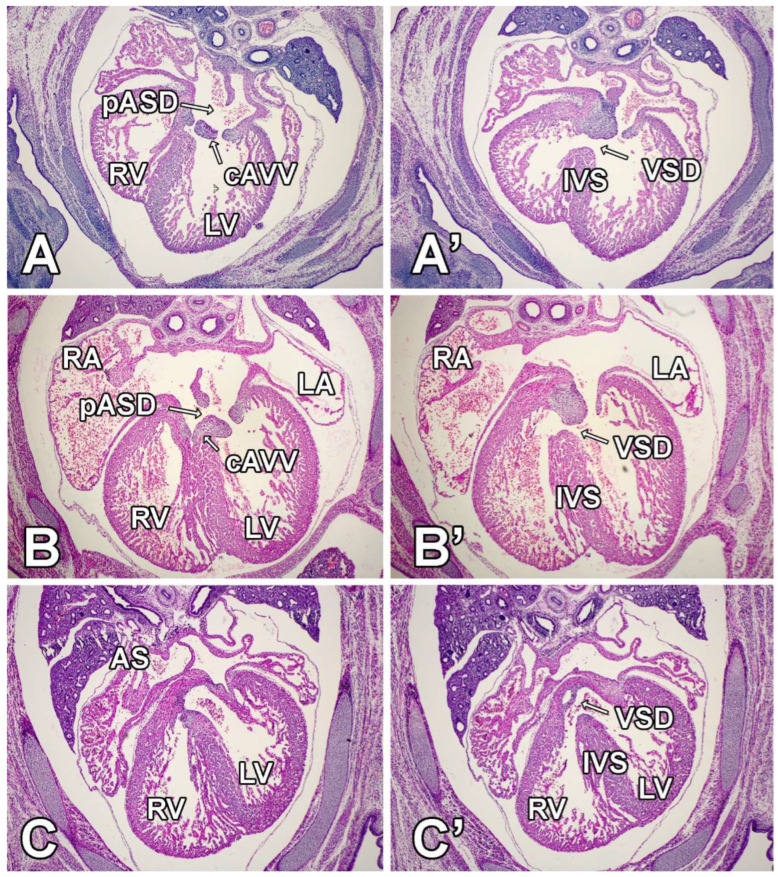
Deletion of Sox9 from the SHF results in atrial and ventricular septal defects. These panels show serial sections of three SHFcre;Sox9fl/fl embryos at ED14.5 (**A**,**A’**), ED15 (**B**,**B’**), and ED14.5 (**C**,**C’**). (**A**,**A’**,**B**,**B’**) show examples of hearts with complete AVSD (primary ASD, common AVV, and inlet type VSD), whereas (**C**,**C’**) shows a heart with an intact atrial septum (**C**) and an isolated subaortic VSD (**C’**). AS = atrial septum, cAVV = common AV valve, IVS = interventricular septum, LA = left atrium, LV = left ventricle, pASD = primary atrial septal defect, RA = right atrium, RV = right ventricle, VSD = ventricular septal defect.

The primary goal of our histological analysis of SHF^cre^;Sox9^fl/fl^;[R26^mG^] specimens was to test the hypothesis that the deletion of Sox9 from the SHF would affect the formation of the AV septal complex. Because the process of atrial and ventricular septation is not completed until ED13.5-ED14.5 [47,48], we restricted our morphological analysis to the 17 SHF^cre^;Sox9^fl/fl^;[R26^mG^] specimens collected at stage ED14.5 or beyond. This analysis identified 16 SHF^cre^;Sox9^fl/fl^;[R26^mG^] specimens with septal defects (Figure 7). Each of these 16 presented with a VSD, while 8 of the 16 also had a pASD. In humans with congenital heart disease, several different forms of VSDs can be distinguished, including inlet VSDs, subaortic VSDs, and muscular VSDs. Classification of defects in humans with congenital heart disease is typically done following guidelines and criteria relevant to the human situation. While muscular VSDs are easy to identify in pre- and postnatal mouse hearts, it is often difficult to assign a single VSD subtype (using criteria for the human) to a heart with complex abnormalities. With that being said, in virtually all of the SHF^cre^;Sox9^fl/fl^;[R26^mG^] hearts in which an pASD was found, the associated VSD spanned between the inlet and outlet. Given the morphology of the AV cushion/valves, we categorized these as complete AVSDs. It is important to note that in most hearts with an inlet VSD, the defect extended into the subaortic region. No valvuloseptal defects were identified in any of the hearts of heterozygote SHF-specific Sox9 knockouts (SHF^cre^;Sox9^fl/+^;[R26^mG^]), or in Sox9^fl/fl^;[R26^mG^] and SHF^cre^;Sox9^+/+^;[R26^mG^] controls.

### 3.5. Deletion of Sox9 from the SHF Does Not Significantly Affect the Initial Development of the DMP and AV Cushions

**Figure 8:**
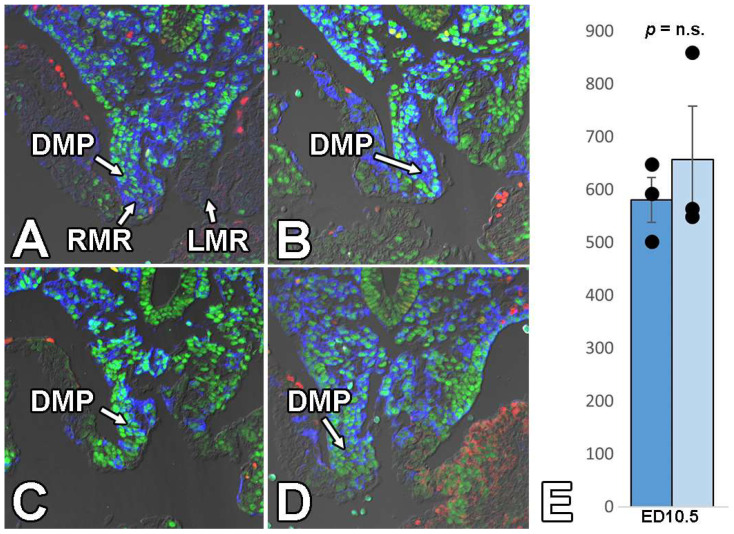
Deletion of Sox9 from the SHF does not affect DMP development. Panels (**A**,**B**) shows the DMP in ED10.5 SHFcre;Sox9fl/+;R26mG control hearts. The sections are stained for Isl1, a transcription factor preferentially expressed in the SHF (green), Sox9 (red), and GFP (blue). Panels (**C**,**D**) show the DMP in SHFcre;Sox9fl/fl;R26mG hearts at ED10.5. The graph in panel (**E**) shows the quantification of the number of cells within the DMP in the controls (dark blue/left) vs. SHFcre;Sox9fl/fl;R26mG hearts (light blue/right). DMP = dorsal mesenchymal protrusion.

Previously we showed that when key players of the BMP and Hh signaling pathways are deleted from the SHF, it leads to perturbation of the development of the DMP and subsequent in pASDs/AVSDs [2,3]. As we found that SHF-specific deletion of Smo resulted in a decrease in the levels of Sox9 in the SHF, we predicted that the analysis of DMP development would reveal that the pathogenesis of pASDs/AVSDs in the SHF^cre^;Sox9^fl/fl^;[R26^mG^] mice was caused by (or at least associated with) hypoplasia of the DMP. The histological assessment and quantitative analysis of the features of the DMP in SHF^cre^;Sox9^fl/fl^;[R26^mG^] embryos at the critical stage of its development (ED10.5) showed, however, that the developing DMP was, by and large, unaffected (Figure 8). Therefore, we inferred that Sox9 in the SHF is not critically important for the initial formation of the DMP and that perturbation of DMP development is not a major contributing factor in the pathogenesis of pASD/AVSDs in the SHF^cre^;Sox9^fl/fl^;[R26^mG^] mouse. 

**Figure 9:**
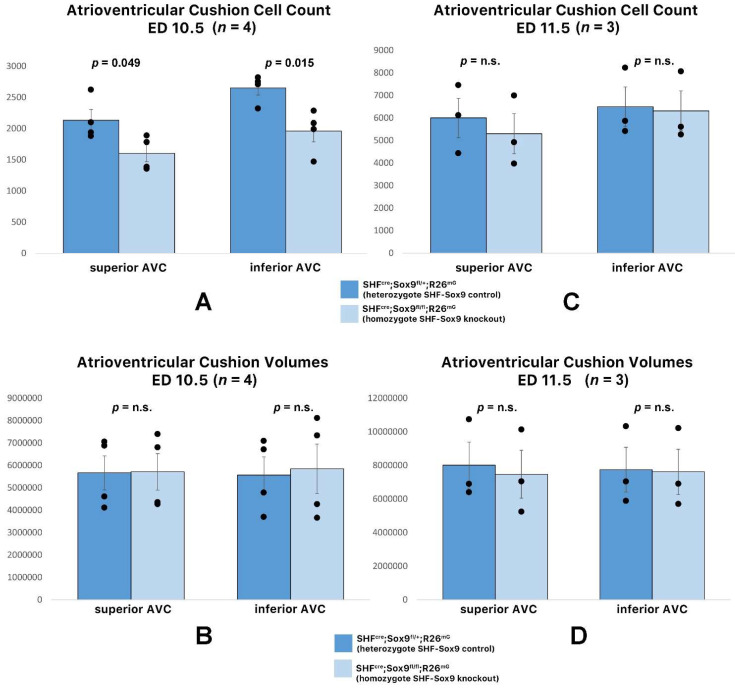
Deletion of Sox9 from the SHF does not significantly affect the development of the AV cushions. Quantitative analysis of the number of cells in, and the volume of, the developing AV cushions of SHFcre;Sox9fl/fl;[R26mG] Sox9 knockouts and heterozygote SHFcre;Sox9fl/+;[R26mG] controls at ED10.5 and ED11.5. The analysis shows a slight reduction in the number of mesenchymal cells in the cushions of the knockouts at ED10.5 (**A**) but no change in the overall volume of the cushions (**B**). The SHF-specific removal of Sox9 has no significant effect on the number of mesenchymal cells in the cushions at ED11.5 (**C**) or on the volume of the cushions at this stage (**D**).

We then turned our attention to the next component of the AV mesenchymal complex: the AV cushions. It is well-established that in early embryonic stages, the mesenchyme of the cushions almost exclusively derives from the endocardium. Lineage analysis with the SHF^cre^;R26^mG^ mouse showed a few GFP labeled cells in the cushions. As Sox9 plays a role in endMT and in the proliferation of ENDCs, it was important to determine whether deleting Sox9 from the SHF would impact cushion development. Quantitative Amira 3D analysis of the developing cushions in SHF^cre^;Sox9^fl/fl^;[R26^mG^] mice and SHF^cre^;Sox9^fl/+^;[R26^mG^] littermates at ED11.5, showed that the volumes were virtually identical (Figure 9). From this we concluded that the deletion of Sox9 from the SHF did not have a significant effect on early AV cushion development. 

### 3.6. Deletion of Sox9 from the SHF Leads to Hypoplasia of the Mesenchymal Cap 

After establishing that the DMP and AV cushions were not likely to play a major role in the pathogenesis of the pASD/AVSDs in the SHF^cre^;Sox9^fl/fl^;[R26^mG^] mice, we moved our focus to the third component of the AV mesenchymal complex, i.e., the mesenchymal cap. In a recent paper, we have shown that the pAS and the associated mesenchymal cap express a series of genes that are also involved in the development of other parts of the AV mesenchymal complex [15]. Although there is still limited insight into how the formation of the cap is regulated (a focus of ongoing studies in the lab), it is well-established that endMT plays an important role in its formation [14,37]. From published data, as well as our own studies using the Tie2^cre^;R26^mG^, Nfatc1^cre^;R26^mG^ and SHF^cre^;R26^mG^ mouse, we conclude that while most of the mesenchymal cells in the cap originate from the First Heart Field (FHF)-derived endocardium, (i.e., GFP negative mesenchyme in SHF^cre^;R26^mG^ specimens), the small numbers of GFP-positive mesenchymal cells seen in the cap of SHF^cre^;R26^mG^ mice could derive from the few GFP-positive endocardial cells that line the cap in SHF^cre^;Sox9^fl/fl^;R26^mG^ hearts. 

**Figure 10:**
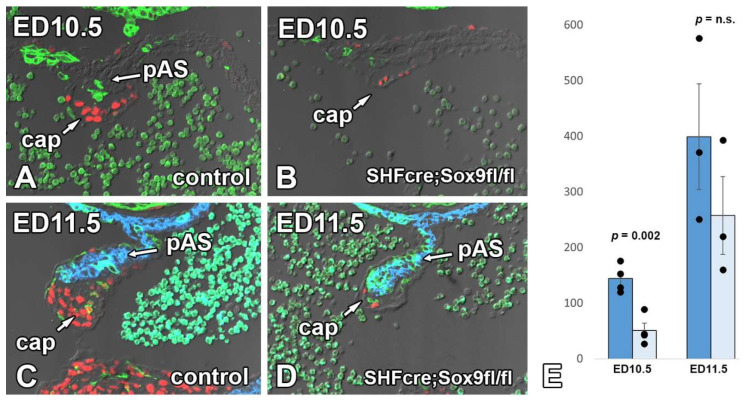
Deletion of Sox9 from the SHF leads to hypoplasia of the mesenchymal cap. Panels (**A**,**C**) show the mesenchymal cap of control hearts at ED10.5 (**A**) and ED11.5 (**C**) whereas panels (**B**,**D**) show the cap in corresponding SHFcre;Sox9fl/fl;[R26mG] littermates. The images demonstrate the significant reduction in size of the cap as well as the reduction in number of mesenchymal cells within the cap. The graph in panel (**E**) shows the decrease in the number of cells as determined by Cell Profiler Analysis in the controls (dark blue/left) vs. SHFcre;Sox9fl/fl;R26mG hearts (light blue/right). The sections in (**A**,**B**) are stained for Sox9 (red) and GFP (green), the sections in (**C**,**D**) for Sox9 (red), GFP (green), and Myosin Heavy Chain (blue). Cap = mesenchymal cap, pAS = primary atrial septum.

The morphological qualitative analysis of individual sections of SHF^cre^;Sox9^fl/fl^;[R26^mG^] and SHF^cre^;Sox9^fl/+^;[R26^mG^] littermates at ED10.5 and ED11.5 showed that the cap in the SHF^cre^;Sox9^fl/fl^;[R26^mG^] mice was considerably smaller than the ones in the heterozygous controls (Figure 10). Subsequent quantitative analysis at ED10.5 and ED11.5 showed a significant decrease both in the number of cells within the cap and the total volume of the cap in the knockout when compared to heterozygous SHF^cre^;Sox9^fl/+^;[R26^mG^] littermates (Figure 10).

Given the established importance of Sox9 for endMT in the AV cushions [31,32,45], we considered the possibility that the deletion of Sox9 from the SHF-derived endocardial cells in the lining of the cap could lead to a specific reduction of SHF-derived mesenchymal cells inside the cap. A quantitative analysis of the cap at ED11.5, did, however, not show a significant difference in reduction of GFP-positive cells in SHF^cre^;Sox9^fl/fl^;R26^mG^ vs. control hearts (15% and 17% respectively), indicating that endMT is not disproportionally affecting SHF-derived endocardial cells over FHF-derived endocardium. We next investigated whether SHF-specific deletion of Sox9 could alter the total number of endocardial cells lining the mesenchymal cap. This analysis, conducted at ED10.5 in control and knockout specimens, revealed a slight reduction (14%) in the total number of endocardial cells in the SHF^cre^;Sox9^fl/fl^;R26^mG^ mouse.

### 3.7. Sox9 Expression in the Endocardial Cell Lineage Is Important for the Development of the Mesenchymal Cap

**Figure 11:**
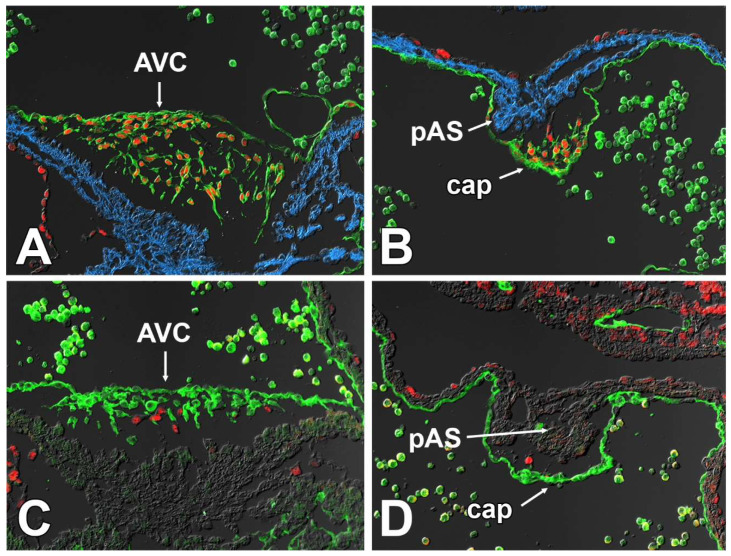
Sox9 expression in the endocardial lineage is critically important for the development of the AV cushions and the mesenchymal cap. Panels (**A**,**B**) show the expression of Sox9 in endocardially derived cells in a control Tie2cre;R26mG specimen at ED10.5 whereas panels (**C**,**D**) show a Tie2cre;Sox9fl/fl;R26mG littermate. Note the absence of Sox9 in the GFPpositive endocardially derived mesenchyme in the hypoplastic AV cushion in (**C**) and the hypoplastic cap in (**D**). AVC = AV cushion, CAP = mesenchymal cap, pAS = primary atrial septum.

The significance of Sox9 in the endocardial cell lineage for AV valve development has previously been shown [29,30,31]. Importantly, these studies also show/report the presence of primary atrial septal defects (pASDs) in Tie2^cre^;Sox9^fl/fl^ specimens. This defect, as mentioned above, is the most common defect in the major forms of AVSDs. Given our observation that the mesenchymal cap is hypoplastic in the SHF^cre^;Sox9^fl/fl^ mouse, and the fact that it has been well-established that the cap is endocardial-derived, we decided to generate Tie2^cre^;Sox9^fl/fl^ mice to determine whether the development of the mesenchymal cap is also affected in this model. Inspection of hearts at E10.5 did indeed reveal significant hypoplasia of the cap (Figure 11). This indicates that the deletion of Sox9 from the endocardial lineage not only affects the AV cushions at the AV junction, but also the development of the cap. Given what is known about the role of Sox9 in the endocardial cell lineage, this could be the result of inhibition of endMT and/or perturbation of events responsible for the expansion of the population of endocardial-derived cells in the cap of Tie2^cre^;Sox9^fl/fl^ mice.

### 3.8. Deletion of Sox9 from the SHF Causes VSDs

**Figure 12:**
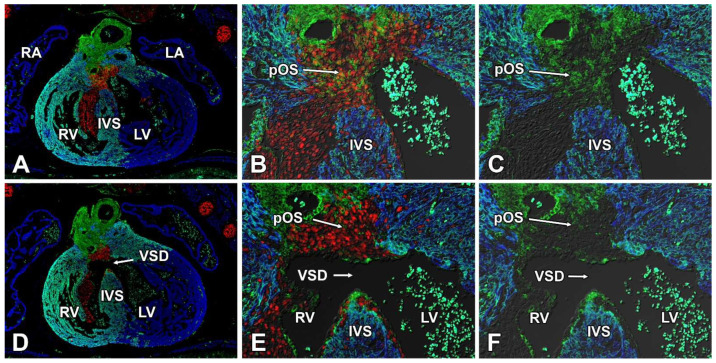
Deletion of Sox9 from the SHF leads to reduced presence of SHF cells in the outlet septum. The sections in this figure were stained for Sox9 (red), Myosin Heavy Chain (blue), and GFP (green). Panel (**A**) shows a control heart at ED14.5 with an intact ventricular septum. Panels (**B**,**C**) show a higher magnification of the junctional area between the proximal outflow tract septum (pOS) and the top of the interventricular septum (IVS). Note the presence of large numbers of Sox9 expressing GFP-positive SHF-derived cells. The image in (**D**) shows a heart of a SHFcre;Sox9fl/fl;[R26mG] specimen with a shortened proximal part of the OFT septum and absence of fusion between the OFT septum with the ventricular septum/AV cushions. Panels (**E**,**F**) also demonstrate the absence of GFP-expressing cells in the pOS. pOS = proximal outflow tract septum, IVS = interventricular septum, LA = left atrium, LV = left ventricle, RA = right atrium, RV = right ventricle, VSD = ventricular septal defect.

Even though the main objective of this study was to determine the role of Sox9 in the posterior SHF in the context of the development of the AV valvuloseptal complex, the high number of VSDs in the SHF^cre^;Sox9^fl/fl^;[R26^mG^] offspring prompted us to look at possible mechanisms on how the deletion of Sox9 from the SHF could result in a VSD. Given the nature of the defect, we focused on the mesenchymal tissues of the outflow tract (OFT), the mesenchyme of the AV cushions, and the muscular ventricular septum. We hypothesized, also based on the descriptive studies by others [46] that these were involved in the pathogenesis of the VSD observed in our system. In the context of this paper, we restricted our analysis on control and SHF^cre^;Sox9^fl/fl^;[R26^mG^] specimens at ED14.5, a stage at which ventricular septation is typically completed. Analysis of serially sectioned and immunofluorescently stained control specimens demonstrated that the proximal OFT septum (formed by the fusion of the OFT cushions), is contiguous with the major AV cushions and the ventricular septum. Importantly, we found that in control hearts, a subset of the mesenchymal cells in the proximal part of the OFT septum is SHF-derived. The analysis of SHF^cre^;Sox9^fl/fl^;[R26^mG^] specimens revealed a shortened proximal part of the OFT septum and and incomplete fusion of the OFT septum with the ventricular septum/AV cushions (Figure 12). The immunofluorescently stained sections illustrated that the proximal OFT septum was largely devoid of SHF-derived cells, suggesting that deleting Sox9 from the SHF affects one or more SHF-derived cell populations that are involved in ventricular septation. Based on the location of the defect, and the fact that the endocardium of the OFT is largely SHF-derived, we hypothesize that this possibly involves a population of endocardial-derived mesenchyme in the proximal outflow tract cushions that has originated from the SHF. Elucidating the mechanism that leads to these VSDs is beyond the scope of this paper but is the focus of an ongoing project.

## 4. Discussion

The AV mesenchymal complex consists of three components; the AV cushions, the DMP, and the mesenchymal cap. Each component needs to develop correctly in order to create the central mesenchymal mass in the middle of the common AV canal which is central to achieving complete AV septation. In earlier papers, we focused most of our attention on the DMP and described how a number of pathways that are active in the Second Heart Field (specifically the BMP, Hh, and Wnt signaling pathways) were found to play a crucial role in DMP development. The data obtained in these studies strongly suggested that the pathogenesis of the AVSDs found in our mouse models was primarily caused by hypoplasia of the SHF-derived DMP. 

Based on the observations discussed earlier in the paper, we had reason to believe that, within the SHF, the transcription factor Sox9 might also play an important role in regulating AV septation. In humans, pathogenic variants of Sox9 can cause campomelic dysplasia (CD), a very serious condition that often results in death of patients in the neonatal period. CD is typically associated with skeletal and facial defects, male-to-female sex reversal, but also with congenital heart malformations, including atrial and ventricular septal defects [49,50,51]. To test the hypothesis that Sox9 expression in the SHF is critically important for AV septation, we created a mouse model in which Sox9 was specifically deleted from the SHF. As expected, approximately, half of all the SHF^cre^;Sox9^fl/fl^ mice developed AVSDs. Unexpectedly, however, we found that all the remaining specimens (with one exception) developed a VSD.

In an effort to determine which element (or elements) of the AV mesenchymal complex was responsible for the AVSDs, we conducted a series of analyses. The analyses of the developing DMP and AV cushions in SHF^cre^;Sox9^fl/fl^;[R26^mG^] embryonic hearts did not show significant abnormalities. Thus, we concluded that the DMP and AV cushions were not major factors in the pathogenesis of pASDs/AVSDs in the SHF^cre^;Sox9^fl/fl^;[R26^mG^] mouse. Our analysis of the mesenchymal cap did, however, reveal a significant reduction in overall size/volume and in the number of cells within the cap. Combined, these results lead us to believe that in the SHF^cre^;Sox9^fl/fl^;[R26^mG^] mouse, hypoplasia of the cap, rather than compromised development of the DMP and/or the AV cushions, is the most likely reason for the observed pASDs/AVSD pathogenesis. 

**Figure 13:**
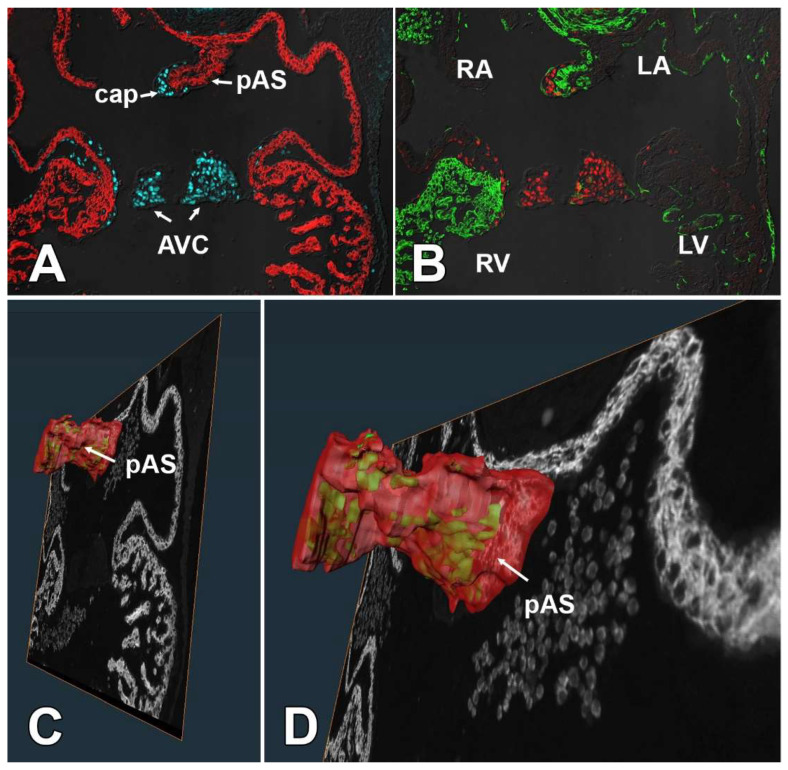
The SHF significantly contributes to the myocardial core of the primary atrial septum. Serial sections of a ED11.5 control heart were stained for Sox9 (blue in (**A)** and red in (**B**)), Myosin Heavy Chain (red in (**A**)) and GFP (green in (**B**)). A 3D rendering of the stained sections was generated in AMIRA software to demonstrate the distribution of the GFP stained SHF-derived cells (green in (**C**,**D**)) within the myocardial core of the primary atrial septum (red in (**C**,**D**)). AVC = atrioventricular cushion, cap = mesenchymal cap, pAS = primary atrial septum, LA = left atrium, LV = left ventricle, RA = right atrium, RV = right ventricle,.

It is well-established that at least the vast majority of the mesenchymal cells in the cap derive from endMT [14,37]. Although very little is known about how endMT of the cap is regulated, literature and preliminary data suggest that BMP and TGF-beta signaling are involved. Additionally, the myocardial core of the primary atrial septum may play a role in the regulation of endMT similar to that of the myocardial AV junction in AV cushion development [16]. A significant reduction in the number of cells in the cap of SHF^cre^;Sox9^fl/fl^;[R26^mG^] embryos can hypothetically be caused by one or more different mechanisms, including reduced endMT, reduced proliferation of the mesenchymal cells, and/or increased levels of apoptosis. In addition, as the population of SHF-derived DMP mesenchymal cells and the endocardial-derived mesenchymal cells in the cap are contiguous (see cartoon), it is theoretically possible that during normal development (some) SHF-derived cells will migrate from the DMP into the cap, a process that could potentially be affected in the SHF^cre^;Sox9^fl/fl^ mouse. The fact that all mesenchymal cells in the cap of Tie2^cre^;Sox9^fl/fl^;R26^mG^ embryos appear to express GFP, indicates that it is very unlikely that this is a major factor in this process. Our data point to inhibition of endMT of the endocardial lining of the cap, rather than a proliferation defect in the cap mesenchyme, increased level of apoptosis, or abnormal migration of SHF-derived cells. The possibility of endMT involvement is supported by observations in the Tie2^cre^;Sox9^fl/fl^ mouse. It is well-documented that in this model deletion of Sox9 from the endocardial cell lineage leads to impaired endMT and subsequent hypoplasia of the AV cushions [29,30,31]. Our own analysis of the Tie2^cre^;Sox9^fl/fl^ mouse showed a significantly reduction in the sized of (and number of cells within) the cap as well, strongly suggesting that endMT of the endocardial lining of the cap is equally dependent on Sox9 expression. The fact that our cell fate tracing studies show that the SHF does not significantly contribute to the endocardial lining of the cap, as well as our observation that both SHF as well as FHF cells are affected inside the cap, leads to the question of what causes the perturbation of endMT in the cap of the SHF^cre^;Sox9^fl/fl^ mouse. As the SHF significantly contributes to the myocardial component of the primary atrial septum (Figure 13), we propose that the deletion of Sox9 in the SHF can have an effect on the formation and differentiation of the pAS which, in turn, could alter the myocardial-mediated regulation of endMT of the endocardial lining of the cap. In ongoing studies, we intend to determine how deletion of Sox9 from the SHF can affect the development of the myocardial core of the primary atrial septum.

Whereas finding a high percentage of AVSDs in the SHF^cre^;Sox9^fl/fl^ offspring was, based on our hypothesis, not surprising (albeit it that the mechanism that led to the phenotype was not as predicted), the fact that all of the SHF^cre^;Sox9^fl/fl^;[R26^mG^] specimens presented with an VSD, either as part of a complete AVSD, or in isolation, was unexpected. The data presented in this paper, as well as other preliminary results obtained in our studies, indicate that deleting Sox9 from the SHF has a profound effect on the contribution of SHF derived cells to a number of different structures in the cardiac outflow tract. A separate manuscript is in preparation that will focus on the role of Sox9 in the SHF as it relates to the development of the outflow tract.

## Data Availability

Not applicable.
